# Advances in the Function Roles of Hydroxycinnamoyl-CoA Shikimate/Quinate Hydroxycinnamoyl Transferases: A Key Enzyme Linking Phenylpropanoid Metabolism to Plant Terrestrial Adaptation

**DOI:** 10.3390/plants15081162

**Published:** 2026-04-09

**Authors:** Jingyi Chen, Chuting Liang, Xian He, Jiayi Huang, Wanying Huang, Anqi Huang, Ying Yang, Gaojie Hong, Yue Chen, Dali Zeng, Jiangfan Guo, Yi He

**Affiliations:** 1 State Key Laboratory for Development and Utilization of Forest Food Resources, Zhejiang A&F University, Hangzhou 311300, China; 19858144707@163.com (J.C.); 15700177407@163.com (C.L.); hjy030606@163.com (J.H.); 19730568810@163.com (W.H.); 13026232531@163.com (A.H.); 2 College of Forestry and Biotechnology, Zhejiang A&F University, Hangzhou 311300, China; 3 College of Agriculture, Nanjing Agricultural University, Nanjing 210095, China; he_xian@stu.njau.edu.cn; 4 College of Modern Agricultural Sciences, Zhejiang Wanli University, Ningbo 315200, China; yingyang@zwu.edu.cn; 5 State Key Laboratory for Quality and Safety of Agro-Products, Institute of Environment, Resource, Soil and Fertilizers, Zhejiang Academy of Agricultural Sciences, Hangzhou 310021, China; gjhong@126.com; 6 Horticulture Research Institute, Zhejiang Academy of Agricultural Sciences, Hangzhou 310021, China; earnchen@163.com; 7 College of Advanced Agricultural Sciences, Zhejiang A&F University, Hangzhou 311300, China; dalizeng@126.com

**Keywords:** hydroxycinnamoyl-CoA shikimate/quinate hydroxycinnamoyl transferase (HCT/HQT), phenylpropanoid pathway, catalytic mechanism, regulatory factors, crop varieties

## Abstract

Hydroxycinnamoyl-CoA shikimate/quinate hydroxycinnamoyl transferase, a key acyltransferase in the phenylpropanoid pathway and a canonical member of the BAHD acyltransferase family (BAHD), catalyzes the formation of pivotal intermediates in the biosynthesis of secondary metabolites such as lignin, chlorogenic acid, and flavonoids. These compounds serve indispensable protective functions in terrestrial plants, underpinning their adaptive responses to abiotic stresses such as drought, ultraviolet (UV) radiation, and oxidative damage. Although the role of HCT/HQT in the core phenylpropanoid pathway has been extensively characterized, its precise functional contributions to the flavonoid biosynthetic branch—particularly with respect to substrate selectivity, kinetic regulation, and metabolic channeling—remain incompletely understood. This review systematically analyzes the structural features, spatial conformation, catalytic mechanism, and substrate promiscuity of HCT/HQT to clarify its molecular determinants of activity and specificity. Furthermore, it highlights regulatory factors influencing HCT/HQT gene expression, such as transcription factors (MYB, bHLH, WRKY), phytohormones (GA_3_, Eth, MeJA, 6-BA, MT), and abiotic/biotic stressors (temperature, blue light, nitric oxide, nano-selenium). Collectively, these insights illuminate how plants dynamically fine-tune phenylpropanoid metabolism in coordination with developmental programs and environmental challenges. This work provides a foundation for further research on HCT/HQT and supports efforts to develop improved crop varieties through targeted regulation of this central metabolic node.

## 1. Introduction

The transition of plants from aquatic to terrestrial environments represents a prolonged and intricate evolutionary process. To withstand adverse terrestrial conditions, including drought, harmful ultraviolet (UV) radiation, loss of buoyancy, broader temperature fluctuations, and exposure to pathogenic microorganisms [1,2], land plants evolved specialized cellular structures that provide protection against these environmental stressors. In angiosperms, such protective barriers are primarily composed of four hydrophobic biopolymers: cutin, suberin, sporopollenin, and lignin [3]. Notably, several precursors of these polymers are biosynthesized via the phenylpropanoid pathway. Within this metabolic pathway, hydroxycinnamoyl-CoA shikimate/quinate hydroxycinnamoyl transferase (HCT/HQT) serves as a key catalytic enzyme in the formation of shikimate and quinate esters—critical intermediates in the biosynthesis of structural biopolymers. HCT/HQT orthologs can display dual enzymatic activity, functioning both as hydroxycinnamoyl-CoA shikimate hydroxycinnamoyl transferase (HCT/HST) and as hydroxycinnamoyl-CoA quinate hydroxycinnamoyl transferase (HQT)—a feature predominantly observed in dicotyledons. In contrast, monocotyledonous HCTs are typically monofunctional [4]. HCT/HQT is a member of the plant BAHD acyltransferase family, a diverse group of enzymes involved in the biosynthesis of secondary metabolites. The acronym ‘BAHD’ is derived from the initial letters of the first four biochemically characterized members in this family [5]: benzyl alcohol O-acetyltransferase (BEAT) [6], anthocyanin O-hydroxycinnamoyl transferase (AHCT), hydroxycinnamoyl/benzoyl transferase (HCBT), and deacetyl vindoline 4-O-acetyltransferase (DAT). Based on protein sequence alignments and phylogenetic analyses, the BAHD acyltransferase family has been classified into five major evolutionary clades (I–V). HCT/HQT belongs to Clade V, representing the most recently diverged subclade within this group [7].

Throughout growth and development, plants biosynthesize a structurally diverse array of secondary metabolites that underpin specialized physiological and ecological functions. However, many of these compounds require post-synthetic modifications—such as glycosylation [8], methylation [9], and acylation [10]—to attain their distinctive functional properties, including lipophilicity, chemical stability, and pharmacological activity [11]. In the post-synthetic modification of plant secondary metabolites, acylation constitutes a pivotal biochemical transformation that imparts essential functional properties to the resulting compounds [7]. The HCT/HQT enzymes, belonging to the BAHD acyltransferase family, catalyze the transfer of acyl groups from acyl-CoA thioesters to hydroxyl or amino groups of acceptor substrates [10], thereby participating in the biosynthesis of important metabolites such as lignin [12], chlorogenic acid [13], and flavonoids [14]. Consequently, HCT and HQT function as central regulatory nodes in the partitioning of phenylpropanoid flux, thereby modulating key agronomic traits such as biomass recalcitrance, stress resilience, and nutritional quality. Functional characterization of these enzymes has advanced progressively—from initial gene cloning and biochemical reconstitution, to systems-level metabolic engineering, and ultimately to contemporary applications in synthetic biology and precision crop improvement. To date, numerous studies have successfully identified and cloned *HCT/HQT* genes encoding hydroxycinnamoyl transferases—namely, *HCT* and *HQT*—from diverse dicotyledonous species, including the medicinal plants dandelion (*Taraxacum antungense*) [15], honeysuckle (*Lonicera japonica*) [16], the model species *Nicotiana benthamiana* [17], and the economically important crops common sunflower (*Helianthus annuus*) [18], tea (*Camellia sinensis*) [19]. Building upon this molecular foundation, researchers have systematically elucidated the regulatory roles of HCT and HQT in phenylpropanoid metabolism through an integrated suite of molecular and functional genomic approaches—including gene cloning and heterologous expression, constitutive and tissue-specific transgenic overexpression, loss-of-function mutagenesis, and quantitative reverse transcription PCR (qRT-PCR)-based expression profiling across developmental stages and under various stress conditions. For instance, in the model legume *Medicago sativa*, targeted downregulation of *HCT* via antisense and RNA interference strategies was shown to reduce lignin deposition by up to 52% while significantly improving cell wall digestibility [20], underscoring its pivotal regulatory role in lignin biosynthesis and cell wall architecture. Heterologous expression of plant HCT/HQT in *Escherichia coli*, supplemented with appropriate substrates, enabled the successful biosynthesis of chlorogenic acid (CGA), thereby demonstrating the functional capability of these enzymes to produce hydroxycinnamoyl conjugates in a microbial system [21]. In sweet potato (*Ipomoea batatas*) calli cultures, overexpression or silencing of the *IbHQTg47130* gene led to differential accumulation of CGA [22]. Moreover, the synthetic biology potential of HCT/HQT has also been demonstrated: transient co-expression of *Crocosmia crocosmiiflora CcHCT* with flavonol glycoside biosynthetic genes in *Nicotiana benthamiana* leaves resulted in a remarkable 30-fold increase in the production of the acylated flavonol glycoside Montbretin A [17]. Collectively, these findings establish *HCT/HQT* as a central regulatory node that governs key agronomic and metabolic traits across plant species—particularly lignification, cell wall recalcitrance, and the biosynthesis of high-value phenolic compounds.

Subcellular localization analyses of HCT/HQT orthologs have revealed taxon-specific distribution patterns across diverse plant lineages. In *Taraxacum antungense*, transient expression of *TaHQT1* and *TaHQT2*—fused to enhanced yellow fluorescent protein (eYFP)—in *Nicotiana tabacum* epidermal cells revealed dual compartmentalization: strong fluorescence signals were observed at the cell periphery, consistent with association with the plasma membrane or apoplastic interface, as well as diffuse cytoplasmic localization and distinct nuclear accumulation. In tomato (*Solanum lycopersicum*), SlHQT was shown to localize to both the vacuolar lumen and the cytoplasmic matrix, indicating potential compartment-specific roles in secondary metabolism [23]. Similarly, in *Lonicera maackii*, distinct subcellular distributions were observed for two closely related enzymes: hydroxycinnamoyl-CoA quinate transferase (LmHQT) was localized exclusively to the cytoplasm, whereas hydroxycinnamoyl-CoA shikimate/quinate transferase (LmHCT) was found predominantly in the nucleus [24]. Collectively, these findings indicate that HCT/HQT orthologs display isoform- and lineage-specific subcellular localization patterns—reflecting spatial compartmentalization of their catalytic activities and suggesting regulation through divergent protein trafficking or targeting mechanisms across plant species.

**Figure 1:**
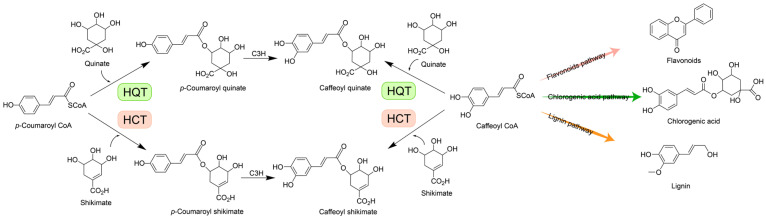
Metabolic roles of hydroxycinnamoyl-CoA: shikimate/quinate hydroxycinnamoyl transferase (HCT/HQT) in phenylpropanoid metabolism. HCT/HQT is a bifunctional acyltransferase belonging to the BAHD family that catalyzes the transfer of hydroxycinnamoyl moieties from hydroxycinnamoyl-CoA donors to either shikimate-yielding *p*-coumaroyl shikimate, the physiological substrate of C3H (*p*-coumaroyl shikimate 3′-hydroxylase), or quinate-yielding chlorogenic acid precursors. Notably, HQT exhibits reversible activity toward both *p*-coumaroyl-CoA and caffeoyl-CoA, producing *p*-coumaroyl quinate and caffeoyl quinate, respectively; similarly, HCT reversibly utilizes the same acyl donors to generate *p*-coumaroyl shikimate and caffeoyl shikimate. The enzymatic function of HCT/HQT is well documented in the biosynthetic pathways of chlorogenic acids and monolignols (precursors of lignin). However, its precise biochemical properties—including substrate selectivity, kinetic parameters, and regulatory mechanisms—within the flavonoid branch of phenylpropanoid metabolism remain incompletely defined. HCT, hydroxycinnamoyl-CoA: shikimate hydroxycinnamoyl transferase; HQT, hydroxycinnamoyl-CoA: quinate hydroxycinnamoyl transferase; C3H, *p*-coumaroyl shikimate 3′-hydroxylase.

Beyond spatial regulation, HCT/HQT orthologs are functionally defined by their essential catalytic roles in the phenylpropanoid pathway—a central metabolic axis governing the biosynthesis of lignin, hydroxycinnamate esters, and flavonoid derivatives. As a pivotal acyltransferase in plant specialized metabolism, HCT/HQT catalyzes the regiospecific transfer of hydroxycinnamoyl groups from hydroxycinnamoyl-CoA thioesters to diverse acceptor substrates, including quinate, shikimate, flavonol glycosides, and anthocyanin aglycones, thereby directing the biosynthesis and tissue-specific accumulation of lignin precursors, chlorogenic acid, and acylated flavonoids [25,26,27,28,29,30]. Silencing of *HCT* in switchgrass (*Panicum virgatum*) resulted in reduced levels of guaiacyl lignin, syringyl lignin, and total lignin, concomitant with a growth-impaired phenotype [12]. Previous studies have identified TaHQT2 as the ultimate rate-limiting enzyme in the chlorogenic acid (CGA) biosynthetic pathway in eastern dandelion (*Taraxacum antungense*) [31]. Furthermore, integrated transcriptomic and metabolomic profiling of Mongolian thyme (*Thymus mongolicus*) identified HCT/HQT orthologs as a central regulatory node within the phenylpropanoid-flavonoid metabolic network. Critically, their enzymatic products, including apigenin, naringenin chalcone, and related apigenin derivatives, have been empirically linked to enhanced tolerance to environmental stress and serve as key determinants of the species-specific volatile aromatic profile that underpins its medicinal and organoleptic properties [32]. Intriguingly, overexpression of *CsHCTs* in transgenic tobacco and Arabidopsis plants was found to increase the accumulation of phenolic acid and lignin while concurrently suppressing the biosynthesis of flavonol glycoside. This implies that *CsHCTs* may redirect carbon flow from the flavonoid pathway toward the biosynthesis of chlorogenic acid, caffeic acid, and lignin, thereby enhancing plant resistance to both biotic and abiotic stresses. Consistent with these findings, CRISPR/Cas9-mediated knockout of the *HQT* gene in tomato (*Solanum lycopersicum*) generated the *SlHQT* mutant lines that completely lacked chlorogenic acid (CGA), while exhibiting significantly elevated levels of hydroxycinnamoyl glucose and flavonoids relative to wild-type plants. Collectively, targeted genetic manipulation of *HCT* and *HQT* orthologs—achieved through transgenic overexpression, CRISPR-Cas9-mediated knockout, or RNA interference-based knockdown—across diverse plant species consistently triggers distinct metabolic reprogramming, characterized by quantifiable alterations in the accumulation of lignin precursors, chlorogenic acid, and acylated flavonoids (Figure 1). These metabolic alterations directly regulate critical physiological traits, including biomass allocation, cell wall integrity, resilience to abiotic stress, and developmental timing.

Current research on HCTs/HQTs has predominantly focused on dicotyledonous plants, with comparatively fewer studies conducted in monocotyledonous and non-vascular plant species. A more comprehensive investigation is therefore warranted to fully elucidate the distribution, functional roles, and evolutionary characteristics of these enzymes across the plant kingdom. Plant metabolic pathways are highly complex, relying on the coordinated action of multiple genes and enzymes. Although HCT/HQT is known to play a role in the biosynthesis of diverse secondary metabolites, our understanding of its precise regulatory mechanisms, particularly across different plant lineages and metabolic contexts, remains limited and warrants further investigation. Extensive global research has been conducted on the structural and functional properties of HCT/HQT, including its regulatory role in plant secondary metabolism, subcellular localization, and functional interactions. This paper presents a systematic review of recent advances in HCT/HQT research, with particular emphasis on their structural characteristics, evolutionary relationships, catalytic mechanisms, and biological functions. Furthermore, we outline key directions for future research, aiming to provide valuable insights and a conceptual framework to guide subsequent studies on HCT/HQT.

## 2. Evolutionary History and Structural Characteristics of HCT/HQT

### 2.1. Evolution Analysis of HCT/HQT

**Figure 2:**
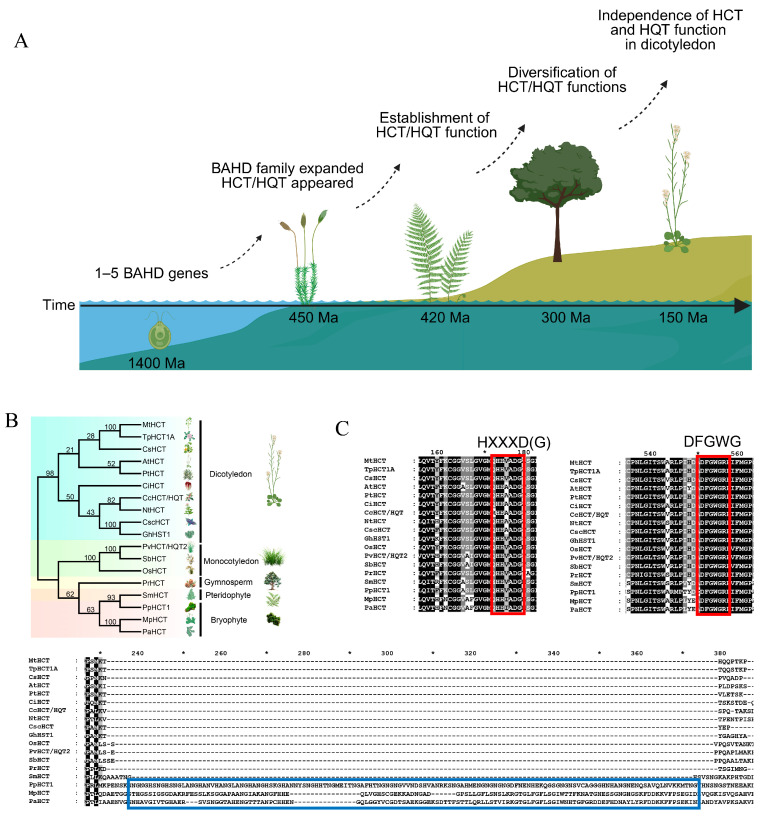
Evolutionary history of hydroxycinnamoyl-CoA: shikimate/quinate hydroxycinnamoyl transferase (HCT/HQT). (**A**) Temporal emergence and diversification of HCT/HQT during the colonization of terrestrial habitats by plants. (**B**) Phylogenetic reconstruction of HCT/HQT proteins from representative land plant lineages. (**C**) Multiple sequence alignment of HCT/HQT orthologs across representative plant species. The aligned sequences include: MtHCT (*Medicago truncatula*, XP_013454529.1), TpHCT1A (*Trifolium pratense*, EU861218), CsHCT (*Cucumis sativus*, JN005932), AtHCT (*Arabidopsis thaliana*, NP_199704.1), PtHCT (*Populus trichocarpa*, XP_006368492.1), CiHCT (*Cichorium intybus*, ANN12608.1), CcHCT/HQT (*Coffea canephora*, ABO47805.1), NtHCT (*Nicotiana tabacum*, AJ507825), CscHCT (*Coleus scutellarioides*, CBI83579.1), GhHST1 (*Glechoma hederacea*, HG423392), OsHCT (*Oryza sativa*, NP_001053225.1), PvHCT/HQT2 (*Panicum virgatum*, AGM90558.1), SbHCT (*Sorghum bicolor*, EES05411.1), PrHCT (*Pinus radiata*, ABO52899.1), SmHCT (*Selaginella moellendorffii*, XP_002979061.1), PpHCT1 (*Physcomitrium patens*, AMK38063.1), MpHCT (*Marchantia paleacea*, MG755753.1), and PaHCT (*Plagiochasma appendiculatum*, MG755754.1). Two evolutionarily conserved acyltransferase motifs, HXXXD(G) and DFGWG, are highlighted in red. A lineage-specific insertion domain, present exclusively in bryophytes and lycophytes but absent in gymnosperms and angiosperms, is shaded in blue. The phylogenetic tree was reconstructed using the neighbor-joining method with 1000 bootstrap replicates in MEGA v11.0.13. Multiple sequence alignment was conducted using MEGA v11.0.13, followed by manual refinement and visualization in GeneDoc v2.7. Ma: Mega—annum, indicates millions of years. The asterisk (*) indicates the scale ruler, marked every 20 amino acid residues.

HCT/HQT is a key enzyme in the phenylpropanoid metabolic pathway and belongs to the BAHD family—a group of plant-specific acyltransferases characterized by the conserved HXXXD motif and DFGWG motif. The earliest BAHD genes were identified in algae, where gene copy numbers remained low (fewer than five). Subsequently, this gene family underwent rapid and extensive expansion during plant evolution, giving rise to multiple phylogenetically distinct clades [33]. *HCT/HQT* orthologs were identified in bryophytes, coinciding with the evolutionary transition from aquatic to terrestrial habitats. In early vascular plants, HCT/HQT was demonstrated to catalyze the biosynthesis of lignin precursors—key structural components that conferred both mechanical support and water-conducting capacity, thereby enabling adaptation to terrestrial environments—and thus established its foundational physiological role in terrestrial colonization. Functional analyses further reveal that approximately 300 million years ago, HCT/HQT acquired enhanced catalytic activity toward substrates involved in chlorogenic acid and flavonoid biosynthesis, indicating a progressive functional diversification during land plant evolution. Subsequently, in dicotyledonous plants, HCT/HQT underwent subfunctionalization, giving rise to a dedicated HQT lineage specialized in the acylation of quinate—reflecting an adaptive refinement of phenylpropanoid metabolism to meet the increasingly complex biochemical demands of terrestrial life (Figure 2A). Like most members of this family, it has a molecular weight of 48–55 kDa, an average amino acid length of approximately 445 residues, and functions as a monomeric enzyme [34]. A total of 18 HCT/HQT sequences—representing both lower and higher plant species—were retrieved from the NCBI database for comparative analysis (Figure 2B). Multiple sequence alignment reveals that HCT/HQT contains two conserved motifs: the HXXXD(G) motif—whose histidine (His) [12] and aspartate (Asp) residues are directly involved in catalysis and collectively form the catalytic core of the enzyme’s active site—and the DFGWG motif, located near the C-terminus. In contrast to the HXXXD(G) motif, the DFGWG motif does not directly participate in catalysis and is primarily located within the C-terminal region of protein [34,35]. Notably, the DFGWG motif displays substantial sequence variability across plant species. For example, in poplar (*Populus*), this motif exhibits substitutions such as DFGWK, DFGFG, and DFGWA [36], whereas in tree peony (*Paeonia ostii*), the PoDPBT enzyme variant carries a DFGGG substitution [37]. Moreover, BAHD acyltransferases involved in flavonoid acylation commonly contain an additional YFGNC motif [38,39]. Site-directed mutagenesis studies have shown that substitutions within these conserved motifs—particularly at one or more functionally critical residues—can markedly alter enzymatic activity [40]. Furthermore, sequence comparisons reveal that homologs from lower plants exhibit substantial similarity to HCT/HQT enzymes from higher plants, yet contain a central insertion exceeding 100 amino acids in their central region—a feature entirely absent in their higher plant counterparts [41] (Figure 2C). This inserted segment may encode regulatory functions that conferred no selective advantage—or were even detrimental to fitness—leading to its loss during evolutionary divergence.

### 2.2. Space Structure of HCT/HQT

**Figure 3:**
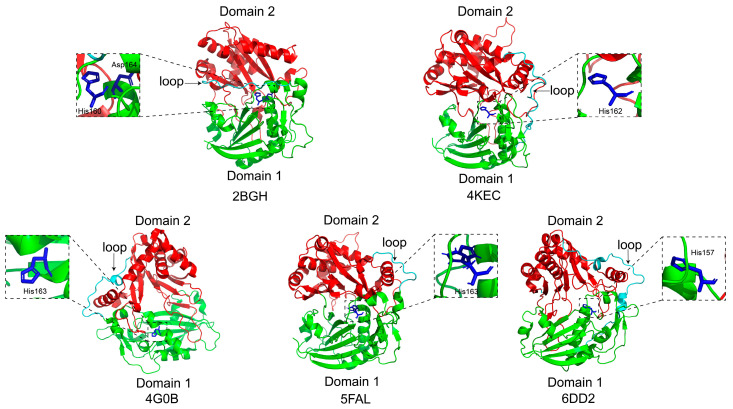
Crystal structures of HCT/HQT and representative BAHD acyltransferases. The HCT/HQT monomer exhibits a two-domain architecture comprising two structurally homologous domains—Domain 1 (green) and Domain 2 (red)—connected by a flexible loop (cyan). Key catalytic residues (blue) are localized within the highlighted active-site pocket. Structural representatives include: 2BGH, vinorine synthase from *Rauvolfia serpentina* (His160 and Asp164); 4KEC, hydroxycinnamoyl-CoA: shikimate hydroxycinnamoyl transferase from *Sorghum bicolor* (His162); 4G0B, hydroxycinnamoyl-CoA: shikimate hydroxycinnamoyl transferase from *Coffea canephora* (His163); 5FAL, hydroxycinnamoyl-CoA: shikimate hydroxycinnamoyl transferase from *Panicum virgatum* (His163); and 6DD2, hydroxycinnamoyl-CoA: shikimate hydroxycinnamoyl transferase from *Selaginella moellendorffii* (His157). All structural coordinates were retrieved from the Protein Data Bank (PDB; https://www.rcsb.org). Molecular visualization and figure generation were carried out using PyMOL v3.0.

The HCT/HQT protein adopts a three-dimensional architecture comprising two structurally distinct domains—Domain I and Domain II—of comparable size, which are connected by an extended crossover loop [34]. In 2012, Lallemand et al. reported the first crystal structure of HCT [33], using vinorine synthase (VS) from *Rauvolfia* (PDB ID: 2BGH) [42] as a search model for molecular replacement (Figure 3). The structure was determined using HCT isolated from *Coffea canephora* (PDB ID: 4G0B) (Figure 3). However, the complex crystal structures of HCT in complex with substrates or acyl-CoA have not been successfully determined, thereby impeding the identification of specific amino acid residues responsible for substrate binding. In 2013, Walker et al. determined the composite crystal structure of HCT in complex with acylated products (sbHCT; PDB ID: 4KEC) from *Sorghum bicolor* [43]. This structure consists of 16 β-sheets and 17 α-helices, with Thr36, Ser38, Tyr40, His162, Arg371, and Thr384 identified as key catalytic residues (Figure 3). In 2016, Eudes et al. reported the crystal structure of the ternary complex HCT from *Panicum virgatum* (PDB ID: 5FAL), comprising PvHCT2a, *p*-coumaroyl-CoA and protocatechuic acid [44]. The substrate-binding site is located at the interface between Domain I and Domain II, where Arg369 forms a stable salt bridge with the substrate’s carboxyl group (Figure 3). In 2018, the crystal structure of SmHCT from *Selaginella moellendorffii* was determined (PDB ID: 6DD2) [45], revealing a fold that is highly conserved among HCT homologs. The active site is located at the interface between two domains and exhibits a cavity volume of 1372.2 ± 10.4 Å^3^. Thr385 and Trp387 were identified as two critical residues involved in both substrate binding and catalysis (Figure 3).

Although substantial progress has been made in elucidating the crystal structure of HCT/HQT enzymes—from isolated apo forms to multi-component complexes—the absence of substrate-bound structures in early studies impeded precise identification of functionally critical residues. Subsequent structural characterization of enzyme–substrate and enzyme–cofactor complexes has progressively overcome this limitation, thereby establishing a robust structural foundation for elucidating the molecular basis of substrate selectivity and the catalytic mechanism of HCT/HQT.

## 3. Catalytic Mechanism of HCT/HQT

The distinctive crystal structures of HCT/HQT—both in their apo forms and in complex with substrates—have provided critical mechanistic insights into the catalytic function of these enzymes. Four evolutionarily conserved amino acid residues—His, Thr, Trp, and Arg—play indispensable roles in catalysis. As members of the BAHD acyltransferase family, HCT/HQT share a catalytic mechanism highly similar to that of vinorine synthase (VS), the first BAHD enzyme for which a detailed catalytic model was established. Specifically, the histidine residue (His160), located within the conserved HXXXD(G) motif at the active site, functions as a general base to deprotonate the O- or N-nucleophile of the acceptor substrate, thereby facilitating a nucleophilic attack on the carbonyl carbon of the CoA thioester. This step generates a tetrahedral oxyanion intermediate that is covalently bound to CoA. Subsequent protonation of this intermediate induces collapse of the tetrahedral adduct, leading to CoA expulsion and formation of the final acylated ester or amide product [42].

Walker et al. (2013) first proposed a detailed catalytic mechanism for SbHCT from *Sorghum bicolor*, based on an integrated structural and kinetic analysis [43]. In this mechanism, His162, assisted by Thr36, abstracts a proton from the C3-hydroxyl group of shikimate, generating a localized oxyanion. This oxyanion subsequently acts as a nucleophilic attack on the carbonyl carbon of the *p*-coumaroyl Coenzyme A (*p*-coumaroyl-CoA) to form a tetrahedral intermediate. The oxyanion of this intermediate is stabilized within a hydrophobic pocket through a hydrogen bond with the indole side chain of Trp386. Subsequent protonation induces collapse of the intermediate, leading to the release of Coenzyme A (HS-CoA) and formation of *p*-coumaroyl shikimate.

Levsh et al. (2016) demonstrated that the N-terminal domain of *Arabidopsis thaliana* AtHCT contains a conserved histidine residue (His153), whose τ-nitrogen acts as a catalytic base to deprotonate the 5-hydroxyl group of the acyl acceptor substrate, shikimate [46]. This deprotonation generates an oxyanion, which serves as a nucleophile to attack the carbonyl carbon of the acyl donor, *p*-coumaroyl-CoA. Concurrently, the carbonyl oxygen of *p*-coumaroyl-CoA forms a hydrogen bond with the indole nitrogen of tryptophan (Trp371) in the C-terminal domain of AtHCT, thereby stabilizing the tetrahedral intermediate formed during catalysis. In the subsequent step, coenzyme A (HS-CoA) is eliminated as a leaving group from the tetrahedral intermediate, affording the acylated product, *p*-coumaroyl shikimate. Additional residues critical for substrate recognition include Thr369 and Arg356: Thr369 forms a hydrogen bond with the 3-hydroxyl group of shikimate, whereas Arg356 engages the shikimate carboxylate group via a salt bridge. Collectively, the acyl transfer reaction catalyzed by HCT/HQT enzymes proceeds through three mechanistically distinct steps: (1) His- and Thr-mediated deprotonation of the acceptor substrate, (2) nucleophilic attack resulting in formation of the tetrahedral intermediate, and (3) transition-state stabilization—achieved through hydrogen bonding (involving Thr369 and Trp371) and electrostatic interactions (mediated by Arg356), ultimately leading to the release of HS-CoA and formation of the acylated product. This evolutionarily conserved catalytic mechanism fundamentally depends on the essential functional contributions of His153, Thr369, Trp371, and Arg356.

Studies have demonstrated that the conformational dynamics of amino acid residue ‘handles’ in HCT/HQT structures are intimately associated with the binding of both the acyl donor and the acyl acceptor substrate. Investigation of five HCT enzymes from diverse plant species revealed a conserved, sub-microsecond ‘swing motion’ of the ‘arginine handle’ across all analyzed structures, fast enough to support efficient substrate recognition under physiological conditions [45]. Notably, the arginine handle (Arg372) in *Selaginella moellendorffii* SmHCT displays distinct structural features compared with other HCTs. Located proximal to the catalytic site, this residue exhibits weak or absent electron density in its side chain—indicative of high conformational flexibility and pronounced spatial protrusion. This unique structural arrangement suggests that Arg372 engages the substrate’s carboxyl group through its positively charged side chain, thereby playing a critical role in substrate recognition across HCT/HQT enzymes. The exceptional conformational flexibility of this arginine residue in SmHCT highlights its potential function as a dynamic molecular switch governing catalytic substrate selection.

**Figure 4:**
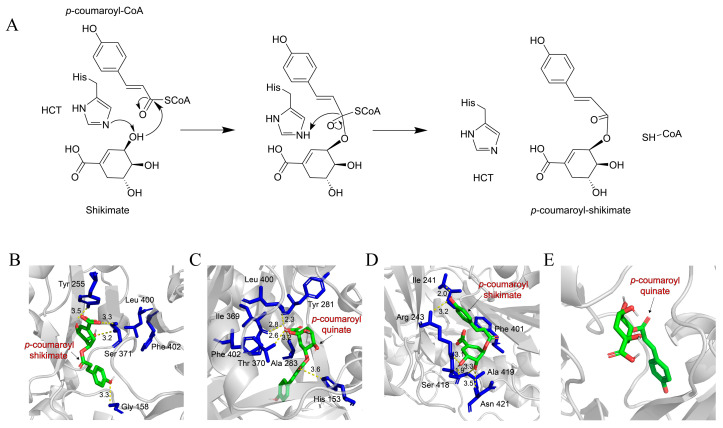
Catalytic mechanism of HCT and comparative active-site architecture of HCT/HQT in dicotyledons and monocotyledons. (**A**) Proposed catalytic mechanism of HCT: a conserved histidine residue deprotonates the C3-hydroxyl group of shikimate, generating an alkoxide nucleophile that attacks the electrophilic carbonyl carbon of *p*-coumaroyl-CoA to form a tetrahedral oxyanion intermediate; subsequent proton transfer to the thiolate leaving group promotes HS-CoA elimination, yielding *p*-coumaroyl shikimate. (**B**) Crystal structure of dicotyledon HCT/HQT in complex with *p*-coumaroyl shikimate. Catalytic residues include Tyr255, Ser371, and Gly158; auxiliary residues contributing to substrate positioning are Leu400 and Phe402. (**C**) Crystal structure of dicotyledon HCT/HQT in complex with *p*-coumaroyl quinate. Catalytic residues include Tyr261, Ile369, Thr370, Ala283, and His153; auxiliary residues are Leu400 and Phe402. (**D**) Crystal structure of monocotyledon HCT/HQT in complex with *p*-coumaroyl shikimate. Catalytic residues include Ile241, Arg243, Ser418, Ala419, and Asn421; the auxiliary residue is Phe401. (**E**) Crystal structure of monocotyledon HCT/HQT in complex with *p*-coumaroyl quinate. No productive binding mode for *p*-coumaroyl quinate is observed—consistent with the absence of detectable HQT activity in monocot HCT/HQT orthologs. Dicotyledon HCT/HQT enzymes exhibit bifunctionality (both HCT and HQT activities), whereas monocotyledon orthologs retain only HCT activity. This functional divergence correlates with substitutions at key positions near the active site, including Leu and Phe residues, that alter substrate specificity and preclude efficient quinate binding. *p*-coumaroyl shikimate and *p*-coumaroyl quinate are shown in green; amino acid residues in blue; oxygen atoms in red; hydrogen bonds as yellow dashed lines.

In summary, the catalytic mechanism of HCT/HQT entails key amino acid residues in the enzyme’s active site abstracting a proton from the acceptor substrate, thereby activating it for nucleophilic attack on the carbonyl carbon of the acyl-donor CoA thioester. Subsequent protonation of the resulting tetrahedral intermediate promotes the departure of coenzyme A (HS-CoA) as a leaving group, affording the acylated product (Figure 4A) [47]. To date, the precise catalytic mechanism of HQT remains uncharacterized. This gap may arise from HQT’s evolutionary origin as a specialized lineage that diverged from the ancestral HCT/HQT clade in dicotyledonous plants—a divergence likely accompanied by adaptive modifications to both its amino acid sequence and three-dimensional structure. Such modifications may perturb the surface electrostatics and hydrophobicity, thereby impeding crystal lattice formation and complicating the identification of viable crystallization conditions. Consequently, structural determination and mechanistic characterization of HQT represent a research direction of considerable scientific importance and strong translational potential.

In this review, molecular docking was employed to simulate and analyze the binding interactions of shikimate and quinate with HCT/HQT enzymes from dicotyledonous and monocotyledonous plants. Structural comparisons revealed pronounced differences in the loop regions surrounding the active site between monocots and dicots. Notably, the active-site cavity of dicot HCT/HQT enzymes exhibits greater conformational flexibility, thereby accommodating a broader spectrum of substrates.

In the docked complexes of dicotyledonous HCT/HQT with *p*-coumaroyl-shikimate (Figure 4B) and monocotyledonous HCT/HQT with *p*-coumaroyl-shikimate (Figure 4D), conserved leucine and phenylalanine residues were identified in proximity to the substrate-binding sites, alongside polar residues, including tyrosine, serine, and threonine. These hydrophobic residues are proposed to stabilize shikimate binding to HCT primarily via favorable van der Waals interactions [33]. Furthermore, a comparative structural analysis was performed between the dicotyledonous HCT/HQT complexes—*p*-coumaroyl-quinate (Figure 4C) and the monocotyledonous HCT/HQT complexes—*p*-coumaroyl-quinate (Figure 4D). The results revealed well-defined binding sites for *p*-coumaroyl-quinate exclusively in the dicotyledonous complexes; no structurally equivalent binding sites were detected in the monocotyledonous homologs. Consistent with this finding, previous genomic studies have reported that no HQT homologs exhibiting significant sequence or functional similarity to dicot HQTs have been identified in monocotyledonous species whose genomes have been fully sequenced [48]. Collectively, these observations support the conclusion that dicotyledonous HCT/HQT enzymes exhibit dual HCT and HQT activities, whereas their monocotyledonous orthologs retain only HCT activity. This functional divergence implies an evolutionary loss of HQT activity in monocots—a phenomenon potentially attributable to structural constraints imposed by conserved leucine and phenylalanine residues proximal to the active site.

## 4. Substrate Promiscuity of HCT/HQT Enzymes

HCT/HQT refers collectively to two distinct enzymatic activities: hydroxycinnamoyl-CoA shikimate hydroxycinnamoyl transferase (HCT, also designated HST) and hydroxycinnamoyl-CoA quinate hydroxycinnamoyl transferase (HQT). The respective acceptor substrates for these enzymes are shikimate and quinate [4]. This review comprehensively summarizes the biochemical properties of HCT/HQT acyltransferases across diverse plant species. The first section of Table 1 lists HCT/HQT enzymes exhibiting dual substrate specificity, i.e., capable of utilizing both shikimate and quinate as acyl acceptors. In contrast, the second and third sections list HCT/HQT enzymes with strict single-substrate preference, utilizing either quinate or shikimate exclusively (Table 1). During evolution, HCT and HQT have diverged to acquire distinct substrate specificities—shikimate for HCT and quinate for HQT. Nevertheless, certain orthologs retain dual catalytic activity, a feature that may contribute to plant adaptability under fluctuating metabolic demands. Structurally, shikimate and quinate differ in their C-1/C-6 moieties: shikimate features a double bond between C-1 and C-6, whereas quinate bears a hydroxyl group at C-1, resulting in distinct three-dimensional conformations for these cyclic polyol compounds. In HCT structures from multiple plant species, the strictly conserved hydrophobic residues Leu400 and Phe402 are positioned adjacent to the C-1 site of the acyl acceptor. Their hydrophobic nature promotes shikimate binding through van der Waals interactions with the C-1=C-6 double bond. By contrast, in HQT enzymes, Leu400 and Phe402 are frequently substituted by Thr and Tyr, respectively—residues capable of forming hydrogen bonds with the C-1 hydroxyl group of the acyl acceptor, thereby enhancing affinity for quinate [33].

HCTs are evolutionarily conserved BAHD acyltransferases that occupy a central position in the phenylpropanoid pathway across all vascular land plants [46,49,50]. These enzymes exhibit low substrate specificity toward both acyl donors and acceptors substrates, enabling them to accommodate a diverse spectrum of substrates through conformational flexibility with their active sites. For instance, Hoffmann et al. (2003) demonstrated that HCT/HQT from tobacco (*Nicotiana tabacum*) can utilize four acyl donors—*p*-coumaroyl-CoA, caffeoyl-CoA, feruloyl-CoA, and sinapoyl-CoA—to synthesize the corresponding acylated ester products, with *p*-coumaroyl-CoA and caffeoyl-CoA exhibiting the highest catalytic efficiency [51]. Although these enzymes exhibit broad substrate tolerance toward acyl donors, they display marked selectivity for acyl acceptors. Sonnante et al. (2010) [35] identified and isolated two hydroxycinnamoyl transferases—*HQT1* and *HQT2*—from artichoke (*Cynara scolymus*) and functionally expressed them in *Escherichia coli* (*E. coli*). Kinetic analyses revealed that both enzymes possess a higher affinity for quinate than for shikimate [35]. In 2011, *CbHST* was cloned from *Coleus blumei* and heterologously expressed in *E. coli*. Subsequent substrate specificity assays revealed that CbHST efficiently transfers diverse acyl groups—including cinnamoyl, *p*-coumaroyl, caffeoyl, feruloyl, and octanoyl—from CoA thioesters to shikimate, but exhibits no activity toward quinate [50]. Moreover, acyl transfer to shikimate proceeds more efficiently than to quinate. Heterologous expression of the full-length *HCT* cDNA from *Cynara cardunculus* in *E. coli* confirmed enzymatic activity with both *p*-coumaroyl-CoA and caffeoyl-CoA as acyl donors. Notably, when quinate serves as the acyl acceptor, the catalytic efficiency of the recombinant enzyme toward *p*-coumaroyl-CoA is 4-fold higher than that toward caffeoyl-CoA [52]. In vitro kinetic analyses by Cardenas et al. [53] demonstrated that NtHCT from tobacco (*Nicotiana tabacum*) preferentially catalyzes the formation of *p*-coumaroyl shikimate from *p*-coumaroyl-CoA and shikimate. Intriguingly, in the presence of free CoA, NtHCT is also capable of catalyzing the reverse reaction. Furthermore, site-directed mutagenesis substituting His153 with alanine (H153A) completely abolishes enzymatic activity. Although NtHCT exhibits a strong preference for shikimate as the acyl acceptor, it also displays measurable activity toward quinate, catalyzing the formation of *p*-coumaroyl quinate under appropriate conditions.

In summary, the HCT/HQT enzyme family displays a distinctive functional architecture: broad substrate tolerance underlies highly selective preferences for acyl acceptors. These enzymes accommodate multiple acyl donors but exhibit evolutionarily conserved, differential specificity toward the two canonical acceptors—shikimate and quinate. While certain family members retain dual-acceptor activity, others have evolved strict specificity for one acceptor over the other. This interplay between functional conservation and plasticity positions HCT/HQT enzymes as pivotal regulatory nodes that govern the metabolic partitioning of lignin precursors and phenolic ester products within the phenylpropanoid pathway.

**Table 1:** Biochemical characteristics of hydroxycinnamoyl-CoA shikimate/quinate hydroxycinnamoyl transferase (HCT/HQT).

Plant Species	NCBI GenBank Accession Numbers	Enzyme	Donor Substrate(s)	Acceptor Substrate(s)	Product(s)	Ref.
*Coffea canephora*	EF137954	HCT/HQT	Caffeoyl-CoA	Shikimate; Quinate	Caffeoyl-shikimate; chlorogenate	[51,54]
*Nicotiana tabacum*	AJ507825	HCT/HQT	Caffeoyl-CoA; *p*-coumaroyl-CoA	Shikimate; Quinate	Hydroxycinnamoyl-shikimate; hydroxycinnamoyl-quinate	[27]
*Nicotiana tabacum*	AJ582651	HQT	Caffeoyl-CoA; *p*-coumaroyl-CoA	Shikimate; Quinate	*p*-coumaroyl-quinate; *p*-coumaroyl-shikimate. Chlorogenate; caffeoyl-shikimate	[55]
*Trifoli* *un pratense*	EU861218	HST	*p*-coumaroyl-CoA; Caffeoyl-CoA	Shikimate; Quinate	*p*-coumaroyl-quinate; chlorogenate	[56]
*Sorghum bicolor*	EES05411	HST	*p*-coumaroyl-CoA; caffeoyl-CoA	Shikimate; quinate	*p*-coumaroyl-shikimate; caffeoyl-shikimate; *p*-coumaroyl-quinate; caffeoyl-quinate	[5,43]
*Morus alba*	MH476577	HCT4	Caffeoyl-CoA; *p*-coumaroyl-CoA	Shikimate; quinate	*p*-coumaroyl-shikimate; caffeoyl-shikimate; *p*-coumaroyl-quinate; caffeoyl-quinate	[29]
*Coffea canephora*	EF153931	HQT	Caffeoyl-CoA	Quinate	chlorogenate	[33,54]
*Cynara cardunculus var. scolymus*	DQ915589, DQ915590	HQT	Caffeoyl-CoA; *p*-coumaroyl-CoA	Quinate	*p*-coumaroyl-quinate; chlorogenate	[57]
*Cynara cardunculus var. scolymus*	EU697935	HQT1	*p*-coumaroyl-CoA; Caffeoyl-CoA	Quinate	*p*-coumaroyl-quinate; chlorogenate	[35]
*Cynara cardunculus var. scolymus*	EU839580	HQT2	*p*-coumaroyl-CoA; Caffeoyl-CoA	Quinate	*p*-coumaroyl-quinate; chlorogenate	[35]
*Nicotiana tabacum*	AJ582651	HQT	*p*-coumaroyl-CoA	Quinate	*p*-coumaroyl-quinate	[53]
*Glechoma* *hederacea*	HG423392	HST1	*p*-coumaroyl-CoA; Caffeoyl-CoA	Shikimate	4-coumaroyl-shikimate; caffeoyl-shikimate	[58]
*Cucumis sativus*	JN005932	HCT	*p*-coumaroyl-CoA	Shikimate	*p*-coumaroyl-Shikimate	[57]
*Populus nigra*	JF693234	HCT1	*p*-coumaroyl-CoA	Shikimate	*p*-coumaroyl-Shikimate	[59]
*Populus tomentosa*	KT021003.1	HCT6	Caffeoyl-CoA	Shikimate	Caffeoyl-Shikimate	[60]
*Camellia sinensis*	XP_028078731	HCT/HQT	Caffeoyl-CoA	Shikimate	Caffeoyl-Shikimate; chlorogenate	[61]
*Populus trichocarpa*	EU603313.1	HCT1	*p*-coumaroyl-CoA; Caffeoyl-CoA	Shikimate	*p*-coumaroyl-shikimate; caffeoyl-shikimate	[62]
*Populus trichocarpa*	EU603314.1	HCT6	*p*-coumaroyl-CoA; Caffeoyl-CoA	Shikimate	*p*-coumaroyl-shikimate; caffeoyl-shikimate	[62]
*Nicotiana tabacum*	AJ507825	HCT	*p*-coumaroyl-CoA	Shikimate	*p*-coumaroyl-shikimate	[53]

Collectively, these findings demonstrate that HCT/HQT enzymes display considerable flexibility in selecting both acyl donors and acceptor substrates. This intrinsic substrate promiscuity holds significant promise for the biocatalytic synthesis of target acylated compounds and esters.

## 5. Functional Characterization of *HCT/HQT* Genes

### 5.1. Involvement of HCT/HQT in Plant Secondary Metabolite Biosynthesis

#### 5.1.1. Lignin Biosynthesis

Lignin is a major structural component of plant cell walls [63] and plays critical roles in plant growth and development [29], defense against pathogen invasion, and resistance to abiotic stress [64]. HCT/HQT enzymes are considered key regulatory components governing both the biosynthesis and polymerization of lignin monomers. By catalyzing the transfer of hydroxycinnamoyl-CoA moieties to specific acceptor molecules, HCT/HQT contributes to the formation of lignin precursors. These monomers subsequently undergo oxidative polymerization to form lignin—a biopolymer that imparts critical mechanical strength to vascular tissues and enables efficient long-distance water transport, thereby facilitating plants’ adaptation, growth and reproductive success in terrestrial environments. The lignin biosynthetic pathway consists of three principal stages [65]. It initiates with the shikimate pathway, wherein photosynthetically fixed carbon is converted into aromatic amino acids—primarily phenylalanine, tyrosine, and tryptophan. This is followed by the phenylpropanoid pathway, in which phenylalanine undergoes deamination and sequential enzymatic modification to yield hydroxycinnamic acids and their corresponding CoA thioesters. Finally, a specialized monolignol-specific pathway converts these hydroxycinnamoyl-CoA esters—through a series of reductions, hydroxylations, and methylations—into monolignols, the direct monomeric precursors of lignin. These monolignols subsequently undergo oxidative polymerization, catalyzed by peroxidase or laccase, to form the heterogeneous, three-dimensional lignin polymer. Throughout this metabolic cascade, HCT/HQT functions as a pivotal regulatory node that directs carbon flux toward lignin biosynthesis.

During the early stages of plant evolution from aquatic to terrestrial habitats, adaptation to gravitational forces and mechanical stresses necessitated the development of structural support systems, such as lignin, to facilitate upright growth [66]. The biochemical function of HCT/HQT has been highly conserved across plant lineages. The *HCT/HQT* gene family first emerged in bryophytes, and orthologs have been identified in early-diverging land plants—including the moss (*Physcomitrella patens*), the liverwort (*Marchantia paleacea*), and the horsetail (*Equisetum arvense*) [41,49,67]. Gene expression profiling and enzymatic assays confirm that these ancestral homologs retain functional properties comparable to those of their vascular plant counterparts and contribute—either directly or indirectly—to monolignol biosynthesis and lignin deposition. To date, numerous studies have successfully cloned and functionally characterized *HCT/HQT* genes from a wide range of angiosperms, including *Paeonia lactiflora*, *Neolamarckia cadamba*, *Populus*, *Medicago sativa*, and *Arabidopsis thaliana*, with functional validation accomplished through heterologous expression and genetic transformation approaches [64,68,69,70,71]. In 2021, Zhao et al. isolated *PlHCT1* from *Paeonia lactiflora* and demonstrated that its overexpression in transgenic tobacco led to a significant increase in lignin accumulation [71]. HCT is recognized as a well-established regulatory enzyme located at a critical metabolic branch point—diverting flux between the lignin and flavonoid biosynthetic pathways. Xia et al. showed that overexpression of *CsHCTs* from *Camellia sinensis* in both tobacco and Arabidopsis resulted in markedly elevated accumulation of phenolic acid and lignin [32]. In 2007, Richard et al. employed antisense RNA-mediated suppression of *HCT* expression in alfalfa (*Medicago sativa*) and observed a substantial reduction in both HCT enzyme activity and lignin content in transgenic lines [72]. Moreover, exogenous hormone treatments have demonstrated that abscisic acid (ABA) [73,74], methyl jasmonate (MeJA), gibberellic acid (GA_3_), salicylic acid (SA) [75], cytokinin (6-BA) [76], and melatonin (MT) [77] can upregulate *HCT* expression—though ABA has been reported to downregulate *HCT* under specific physiological or experimental conditions, underscoring the context-dependent nature of its regulatory effects. Collectively, these regulatory mechanisms fine-tune lignin deposition and exert profound effects on plant growth, development, and stress responses. Together, these findings establish a critical theoretical foundation for the functional characterization of *HCT/HQT* genes and their biotechnological applications in crop improvement.

#### 5.1.2. Chlorogenic Acid Biosynthesis

Chlorogenic acid (CGA) is a pivotal defensive phytochemical that has evolved in plants as a critical component of their long-term adaptive strategy, conferring enhanced tolerance to both biotic and abiotic stresses. It is a soluble phenolic secondary metabolite biosynthesized via the phenylpropanoid pathway under aerobic conditions [78] and represents one of the most abundant and bioactive natural compounds. A growing body of evidence demonstrates that CGA plays a pivotal role in mediating plant responses to diverse environmental stresses [33]. During the evolutionary divergence of plant species, the CGA biosynthetic pathway has likely undergone adaptive modifications to meet species-specific physiological and ecological requirements under distinct environmental conditions. For instance, in dicotyledonous plants such as potato (*Solanum tuberosum*) [78] and honeysuckle (*Lonicera japonica*) [79], CGA is the most abundant phenolic antioxidant—potentially reflecting the elevated oxidative stress experienced by dicots in their native habitats. In these species, the expression and regulation of *HCT/HQT* genes appear to have undergone evolutionary fine-tuning to sustain high-level CGA accumulation, thereby enhancing plant resilience against oxidative damage. *p*-coumaroyl-CoA serves as a central precursor in CGA biosynthesis and is specifically channeled by enzymes including quinate hydroxycinnamoyl transferase (HQT), shikimate/quinate hydroxycinnamoyl transferase (HCT) [80] and *p*-coumaroyl ester 3′-hydroxylase (C3′H). To date, research on chlorogenic acid (CGA) has primarily centered on its chemical properties, while its genetic and molecular regulatory mechanism has received comparatively limited attention [81]. Zhao et al. (2019) [30] performed transcriptomic analyses of key genes involved in the CGA biosynthesis pathway in mulberry trees (*Morus alba*), revealing a significant positive correlation between *HCT* gene expression and CGA accumulation. Subsequent enzymatic assays confirmed that MaHCT4 efficiently catalyzes the formation of *p*-coumaroyl shikimate, *p*-coumaroyl quinate and CGA [30]. Similarly, Cadena-Zamudio et al. (2020) used hornwort (*Ceratophyllum demersum*) as an experimental system and, through transcriptome profiling under nitrate-deficient conditions, identified HCT as a key enzyme in the CGA biosynthetic pathway [4]. Collectively, these transcriptomic studies establish a foundational framework for elucidating the biosynthetic mechanism of CGA and for characterizing the transcriptional and post-transcriptional regulatory networks that govern *HCT*-related genes in plants.

#### 5.1.3. Flavonoid Biosynthesis

Flavonoids present a major class of natural plant secondary metabolites, characterized by a distinctive 15-carbon skeleton [82]. Their core structure adopts a C6-C3-C6 framework, consisting of two aromatic rings (designated A and B) connected by a three-carbon bridge that undergoes cyclization to form an oxygen-containing heterocyclic ring (C) [83]. This highly conserved structural architecture has been evolutionarily refined over extended geological timescales and serves as a define structural motif that unifies the flavonoid family.

During plant evolution, flavonoids have diversified into a broad spectrum of structural subclasses, including anthocyanins, flavones, flavonols, flavanones, dihydroflavonols, chalcones, aurones, flavanes-3-ols (and their oligomeric and polymeric forms, proanthocyanidins) and isoflavones [82]. Among these, anthocyanins—water-soluble flavonoid pigments widely distributed across higher plants—play a pivotal role in plant adaptation and evolutionary success. From an evolutionary perspective, they not only impart diverse colors to plants tissues—thereby attracting pollinators and facilitating sexual reproduction—but also enhance fitness under natural selection [84]. Moreover, anthocyanins exhibit potent antioxidant and anti-inflammatory activities, enabling plants to mitigate oxidative stress and enhance resistance to pathogen invasion. Collectively, these functions improve plant adaptability to complex environmental stresses and underscore the substantial research significance of anthocyanins. In contrast, the flavonoid biosynthetic pathway in lower plants relies predominantly on chalcone synthase (CHS) and chalcone isomerase (CHI), whereas the functional roles of hydroxycinnamoyl transferase (HCT) and hydroxycinnamoyl quinate transferase (HQT) remain unclear [85]. In contrast, research on the roles of HCT/HQT in flavonoid metabolism in higher plants remain limited. Saophea et al. ectopically overexpressed *Arabidopsis thaliana* production of anthocyanin pigment 1 (AtPAP1)—a master transcription factor regulating anthocyanin biosynthesis, in *Solanum nigrum*. They observed concomitant upregulation of several core flavonoid pathway genes, including phenylalanine ammonia-lyase (PAL) [35,85], cinnamic acid-4-hydroxylase (C4H), and hydroxycinnamoyl-CoA transferase (HCT)—alongside significantly enhanced anthocyanin accumulation in transgenic plants. Using a heterologous expression system in *Solanum nigrum*, Chhon et al. (2020) further demonstrated that overexpression of *AtPAP1* activates anthocyanin biosynthesis through the evolutionarily conserved plant flavonoid pathway [86]. Moreover, Li et al. (2022) integrated multi-omics analyses with qRT-PCR validation in peanut (*Arachis hypogaea*) and identified *Ah21440*—a gene encoding a hydroxycinnamoyl transferase (HCT)—whose expression is significantly correlated with anthocyanin biosynthesis [87].

The findings provide valuable insights into the metabolic function of HCT in anthocyanin biosynthesis in peanut, thereby enhancing our understanding of how regulatory genes orchestrate the expression of key biosynthetic enzymes during plant evolution. This knowledge may aid in elucidating the mechanisms by which plants dynamically regulate anthocyanin metabolism to respond to environmental stresses and fulfill developmental and growth requirements.

### 5.2. Involvement of HCT/HQT in Plant Defense Mechanisms

Hydroxycinnamic acid amides (HCAAs), synthesized through the enzymatic activity of HCT or HQT [88], form a complex, cross-linked network by covalently binding to cell wall components. This structural reinforcement enhances mechanical strength and imparts resistance to pathogen invasion. In *Camellia sinensis*, transient overexpression of *CsHCTs* enhances the biosynthesis of the defense-related compound epicatechin-3-O-caffeoate (EC-CA), thereby significantly mitigating pest-induced damage to tea leaves [19]. Moreover, upon pathogen infection, *HCT/HQT* genes—key regulators of plant immunity—are rapidly upregulated, playing a critical role in disease resistance [89,90]. In tomato plants inoculated with Pcc (*Pectobacterium carotovorum* subsp. *carotovorum*), quantitative analysis of defense-related genes, including those encoding cytochrome P450 enzymes, chitinase, and HCT, revealed time-dependent differential expression at 24 and 72 h post-inoculation, collectively contributing to resistance against Pcc [91].

Abiotic stress can activate immune signaling pathways, thereby enhancing overall stress tolerance. Under salt stress, proteomic analysis of xylem sap in *Brassica oleracea* revealed significant accumulation of HCT—a key enzyme involved in the stress responses—concomitant with enhanced xylem differentiation and lignification [92]. Under low-temperature stress, *HCT* expression is upregulated in tobacco, leading to increased accumulation of polyphenol, particularly lignin, which confers protective benefits. Enhanced lignin biosynthesis strengthens cell wall mechanical integrity and established robust chemical defense barriers, thereby effectively safeguarding plants against both biotic pathogens and abiotic environmental challenges [93].

Plant hormone levels also modulate the expression of *HCT/HQT*, thereby significantly influencing resistance to specific pathogens. Abdelkhalek et al. (2020) demonstrated that the treatment of the upper halves of *Chenopodium amaranticolor* leaves with 200 µg/mL ethanol extract of the whole plant (WPE) of *Haplophyllum tuberculatum*—applied either 24 h before or after viral inoculation—markedly reduced local lesion formation [94]. This treatment significantly upregulated transcript levels of pathogenesis-related protein-1 (*PR-1*), chalcone synthase (*CHS*), and *HQT* genes, thereby enhancing resistance against *Fusarium* spp., *Rhizoctonia solani*, and tobacco mosaic virus (TMV). In contrast, jasmonic acid (JA), salicylic acid (SA), and ABA have been shown to upregulate HQT expression, thereby improving resistance to insect herbivory [26].

In summary, HCT/HQT enhances plant defense through multiple coordinated mechanisms: (i) the biosynthesis of defense-related secondary metabolites, (ii) reinforcement of cell wall integrity, and (iii) activation of immune signaling pathways. Moreover, the expression of HCT/HQT is tightly regulated by hormonal signals, enabling dynamic, stress-responsive control over the production of defensive secondary metabolites—thereby enhancing plant resilience to both biotic and abiotic stresses.

### 5.3. Applications of HCT/HQT in Agricultural Production

With advances in synthetic biology technologies, numerous structurally complex natural compounds have been successfully biosynthesized in engineered microbial hosts—such as *Escherichia coli* [52] and *Saccharomyces cerevisiae* [17]—and substantial progress has also been made in synthetic biology research on HCTs/HQTs.

In recent years, significant progress has been achieved in the agricultural applications of HCTs/HQTs, primarily in three areas.

In the bioenergy sector, significant reductions in lignin content have been achieved through suppression of *HCT/HQT* genes expression. Lignin contributes to structural integrity, mechanical strength, and hydrophobicity to secondary cell walls; however, it also hinders the penetration of hydrolytic enzymes into polysaccharide substrates. This limitation impairs the efficient separation of cellulose fibers and hinders the enzymatic hydrolysis of cellulose and hemicellulose into fermentable sugars during biorefining processes [64]. Notably, Tong et al. (2015) developed a novel genetically modified alfalfa line with enhanced biofuel potential by simultaneously downregulating two key enzymes—HCT and *p*-coumaroyl ester 3′-hydroxylase (C3′H)—in the lignin biosynthetic pathway, thereby reducing lignin content and increasing cellulose accumulation [20]. Suppression of HCT expression resulted in a substantial decrease in lignin levels (up to 40% reduction) and significantly improved dry matter digestibility (by up to 20%), thereby improving both forage quality and saccharification efficiency for bioethanol production [63,71,72]. Eudes et al. (2016) [44] exploited the substrate promiscuity of HCT through metabolic engineering to enhance its catalytic activity toward non-natural substrates. This intervention impaired the biosynthesis of coniferyl and sinapyl alcohols, thereby reducing lignin deposition, and improving the enzymatic saccharification efficiency of lignocellulosic biomass [44]. In woody species, HCT plays a critical role in modulating cell wall mechanics. Transgenic poplar lines with down-regulated HCT expression exhibited smaller fiber cells and vessel elements, significantly reduced cell wall density, and a 7.0% increase in modulus of elasticity (MOE) relative to non-transgenic control [71], thereby presenting a promising strategy for enhancing wood mechanical properties.

In crop quality improvement, the application of HCT/HQT has expanded from forage crops to postharvest fruit preservation. Wang et al. (2024) demonstrated that postharvest treatment of passion fruit with aloe vera (ALV) and tea polyphenols [10] upregulates transcript levels of key phenylpropanoid pathway genes—including *PeC4H* and *PeHCT*-thereby promoting the accumulation of beneficial phenolic acids and flavonoids and enhancing postharvest fruit quality [95].

In high-value natural product synthesis, microbial platforms based on HCTs/HQTs have achieved significant breakthrough. By systematically engineering the glucose metabolic pathway in *E. coli* and heterologously expressing HQT, a de novo biosynthetic route for CGA was successfully established. This strategy achieved an estimated 35–40% reduction in production cost compared with conventional chemical synthesis starting from caffeic acid [96]. Kim et al. (2013) further enhanced the shikimate pathway in *E.coli* through co-overexpression of tobacco-derived shikimate hydroxycinnamoyl transferase (*NtHST*) and rice-derived 4-coumarate, CoA ligase (*4CL*), thereby establishing a robust microbial platform for the production of hydroxycinnamic acid derivatives—including *p*-coumaroyl shikimate ester and CGA [21].

In addition, chlorogenic acid (CGA), a valuable secondary metabolite synthesized through HCT-mediated pathways, exhibits potent antioxidant and anti-inflammatory properties and has demonstrated preventive efficacy against a range of diseases [97]. Liu et al. (2019) [15] identified that the *TaHQT1* and *TaHQT2* genes regulate the biosynthesis of 5-caffeoylquinate—a key precursor of CGA-in dandelion (*Taraxacum officinale*). These findings offer promising genetic strategies to enhance the accumulation of 5-caffeoylquinate in plants for medicinal or nutraceutical applications.

Owing to its distinctive structural features and broad substrate tolerance, HCT/HQT has emerged as a versatile biocatalyst with wide-ranging applications in bioenergy development, crop improvement, and the production of antioxidant and anti-inflammatory pharmaceuticals.

Despite its promising prospects, the widespread agricultural application of HCT/HQT faces multiple limitations:

The first challenge stems from biological limitation inherent to the complexity and robustness of plant metabolic systems. Functional redundancy within the BAHD family implies that down-regulating a single *HCT* or *HQT* gene may be compensated by other homologous genes, thereby diminishing the intended regulatory effect. More critically, inhibiting lignin synthesis may trigger a redistribution of carbon flux toward alternative metabolic pathways, resulting in unintended metabolic perturbations. Moreover, functional divergence of *HCT* and *HQT* between dicotyledonous and monocotyledonous species necessitates species-specific regulatory strategies. To overcome this biological limitation, multi-gene editing—targeting not only *HCT* and its homologs but also upstream transcription factors such as MYB and NAC—should be integrated with comprehensive metabolic flux analysis to enable precise, directed remodeling of carbon allocation.

The other challenge is translational limitation—the difficulty of scaling discoveries from controlled laboratory or greenhouse environments to real-world field conditions. Findings obtained under greenhouse environments often fail to replicate in field trials. For example, although reducing lignin content improves biomass conversion efficiency, it may simultaneously compromise key agronomic traits such as lodging resistance and disease resistance, thereby complicating the optimization of multiple performance attributes. Moreover, the commercialization of genetically modified crops continues to be impeded by stringent regulatory requirements and enduring public skepticism. To mitigate translational limitation, priority should be given to employing tissue-specific or chemically inducible promoters. Such promoters enable precise spatiotemporal control of transgene expression—restricting regulatory effects to specific tissues or developmental stages—and thereby help preserve overall stress resilience.

## 6. Regulatory Factors Influencing *HCT/HQT* Gene Expression

HCT/HQT is a pivotal enzyme in the plant phenylpropanoid pathway, and its expression is tightly regulated by a diverse array of endogenous and exogenous factors, including transcription factors, phytohormones, and abiotic and biotic stresses. These regulatory signals integrate within a complex, multilayered network that orchestrates the spatiotemporal expression of *HCT/HQT* during secondary metabolism, thereby fine-tuning the biosynthesis of critical metabolites such as lignin and chlorogenic acid.

### 6.1. Transcription Factor

The MYB transcription factor (TF) family—one of the largest TF families in plants—plays a pivotal role in diverse biological processes, including plant growth and development, cell morphogenesis, tissue patterning, primary and secondary metabolism, and responses to environmental stresses. *MYB8* was isolated from tobacco leaves, and its expression was suppressed using virus-induced gene silencing (VIGS). In *MYB8*-silenced plants, the transcript level of *HCT-LIKE* was markedly reduced, concomitant with stem softening and decreased lignin content [98]. Wang et al. (2017) [99] cloned *CmMYB19* from *Chrysanthemum morifolium* and demonstrated, through yeast one-hybrid assays, that CmMYB19 specifically binds to the AC cis-element in the promoters of genes involved in lignin biosynthesis. Transgenic chrysanthemum lines overexpressing *CmMYB19* exhibited significant upregulation of key phenylpropanoid pathway genes, including *PAL1*, *C4H*, and *HCT* [99]. Payyavula et al. (2014) transiently overexpressed the potato MYB transcription factor StAN1 in tobacco and observed increased activity of *HQT*—a key gene in CGA biosynthesis—along with increased CGA accumulation [27]. Rommens et al. (2008) demonstrated that specific expression of *StMtf1M* activates the phenylpropanoid pathway, including upregulation of *HQT* expression [100]. In Dandong dandelion (*Taraxacum antungense*), TabHLH1 directly binds to the bHLH-binding motif in the *proTaHQT2* promoter. Overexpression and RNA interference (RNAi)-mediated knockdown of *TabHLH1* modulated CGA and luteolin accumulation by upregulating or downregulating the expression of *TaHQT2* and *Ta4CL*, respectively [31]. Furthermore, WRKY transcription factors have also been implicated in the regulation of *HCT* expression. Zhang et al. (2008) integrated a genome-wide association study (GWAS) with expression quantitative trait loci (eQTL) analysis and identified that the *PtHCT2* expression in poplar (*Populus*) leaves is governed by cis-acting eQTLs containing W-box motifs—the canonical DNA-binding sites for WRKY transcription factors [101]. Collectively, these findings demonstrate that transcription factors—including MYB, bHLH, and WRKY—directly or indirectly regulate *HCT/HQT* and associated pathway genes (e.g., *PAL* and *4CL*), thereby fine-tuning the biosynthesis of plant secondary metabolites. This regulatory network provides both a theoretical foundation and an experimental basis for crop improvement—such as reducing lignin content to enhance forage digestibility—as well as for increasing the accumulation of bioactive compounds (e.g., chlorogenic acid) in medicinal plants.

MYB and bHLH transcription factors associate with WD40 proteins to form MYB-bHLH-WD40 (MBW) complexes, which act synergistically to activate genes encoding enzymes in the phenylpropanoid pathway [102]. For instance, in *Arabidopsis thaliana*, MYB123 and bHLH3 physically interact to form a functional complex that coordinately regulates proanthocyanidin biosynthesis [103]. However, whether MBW complexes directly regulate *HCT* or *HQT* expression remains to be experimentally validated.

### 6.2. Phytohormones

Phytohormones orchestrate plant growth, development, and metabolic processes, and can also act as key regulators of flavonoid biosynthesis. Jie et al. (2022) investigated the effects of exogenous gibberellin (GA_3_) and ethylene application on *Boehmeria nivea*, demonstrating that co-treatment with GA_3_ and Eth significantly reduced lignin content in both leaves and stems [104]. Concurrently, flavonoid accumulation increased, whereas expression of *HCT* genes was downregulated in the treated tissues. Mechanistically, GA and ethylene signaling pathways may converge through shared transcriptional regulators—particularly ethylene-responsive factor (ERF) proteins and DELLA repressors. Accumulating evidence indicates that DELLA proteins physically interact with group VII ERF transcription factors, forming a critical integration node in GA-ethylene crosstalk. Notably, ERF11 has been shown to simultaneously suppress ethylene biosynthesis and promote GA signaling by directly antagonizing DELLA activity [73,105]. This integrated regulatory network likely orchestrates the transcriptional regulation of downstream *HCT/HQT* genes. In parallel, Liu et al. (2022) [88] treated tea plants (*Camellia sinensis*) with methyl jasmonate (MeJA) and the synthetic cytokinin 6-benzylaminopurine (6-BA), and observed a significant correlation between leaf lignification and the expression levels of *CsHCT* genes. These findings suggest that MeJA and 6-BA exert opposing regulatory effects on lignin deposition in tea leaves—potentially through differential modulation of *CsHCT* expression [76]. Zhu et al. (2024) applied melatonin (MT) to stem wounds on melon scions grafted onto pumpkin rootstocks and observed a significant increase in HCT enzyme activity and markedly enhanced lignin deposition, thereby accelerating wound healing in the grafted stems [77]. Despite these advances, the molecular mechanisms governing hormone-mediated regulation of *HCT/HQT* remain poorly characterized. For instance, it remains unclear which transcription factors—such as members of the MYB, WRKY, or NAC families—mediate hormonal signals (e.g., GA or MeJA) to modulate *HCT/HQT* expression, or whether hormone-responsive cis-regulatory elements are present in the promoters of these genes.

### 6.3. Abiotic and Biotic Stresses

Temperature exerts a significant influence on the expression of genes involved in plant secondary metabolism, thereby modulating the biosynthesis of secondary metabolites and enhancing plant adaptation to fluctuating environmental conditions [106]. Sun et al. (2018) employed qRT-PCR to examine seasonal variations in *CsHCT* transcript abundance in *Camellia sinensis* leaf tissues and observed a significant negative correlation between *CsHCT* expression levels and ambient temperature [74]. During cold-induced lignification, bHLH and MYB transcription factors have been demonstrated to form functional protein complexes that cooperatively regulate the expression of phenylpropanoid pathway genes—thereby implicating a temperature-responsive transcription factor signaling axis. However, direct transcriptional regulation of *HCT/HQT* by MYB/bHLH complexes remains to be experimentally validated [107]. Furthermore, exogenous application of nitric oxide (NO) and sodium selenite (Na_2_SeO_3_) significantly upregulated the expression of *HCT1* and *HQT1* in *Cichorium intybus*, elevating transcript levels by approximately 30-fold and 25-fold, respectively [108]. Light is another critical environmental cue that regulates the biosynthesis and accumulation of plant metabolites. Xie et al. (2021) [109] demonstrated that blue light exposure enhances the synthesis and accumulation of multiple bioactive compounds—including chlorogenic acid, rosmarinic acid, quercitrin, and quercetin—in the root tissues of *Sarcandra glabra*. Transcriptomic profiling revealed that this enhancement coincides with the upregulation of *HCT* [109].

**Figure 5:**
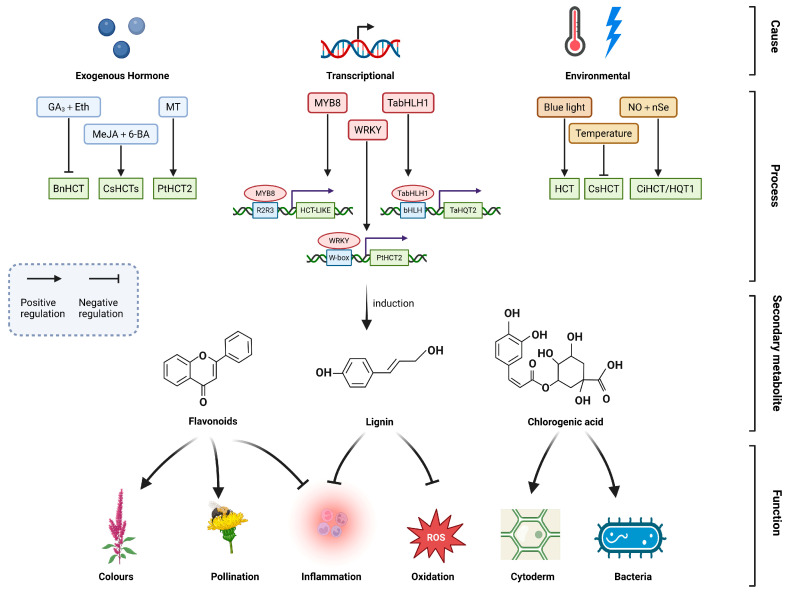
Regulatory network controlling *HCT/HQT* gene expression and physiological roles of HCT/HQT enzymes in plants. Transcriptional regulation of *HCT/HQT* genes is orchestrated by extrinsic environmental cues, including temperature, light quality (particularly blue light), nitric oxide (NO), nano-selenium (nSe), melatonin (MT), and phytohormones, as well as intrinsic transcription factors, predominantly from the MYB, bHLH, and WRKY families. Temperature exerts a negative regulatory effect on *HCT/HQT* transcript accumulation. In contrast, blue light exposure induces transcriptional activation of *HCT/HQT* genes. NO, nSe, methyl jasmonate (MeJA), and 6-benzylaminopurine (6-BA) act as positive regulators of HCT/HQT expression, whereas the phytohormone combination gibberellic acid (GA_3_) and ethylene [104] suppresses *HCT* transcription. MYB, bHLH, and WRKY transcription factors enhance HCT/HQT expression by directly binding to cognate cis-regulatory elements in their promoter regions. Functionally, HCT/HQT enzymes catalyze essential acyl transfer reactions in the biosynthesis of flavonoids, chlorogenic acid, and lignin, three structurally and physiologically distinct classes of plant secondary metabolites. Flavonoids mediate pollinator attraction through floral pigmentation and, together with chlorogenic acid, confer antioxidant and antimicrobial protection against oxidative stress and pathogen infection. Lignin provides structural integrity to plant cell walls, underpinning mechanical support, biotic defense, and tolerance to abiotic stresses. Symbols: ‘→’ denotes activation; ‘⊣’ denotes repression; ‘↱’ denotes the direction of gene transcription. GA_3_, gibberellic acid 3; Eth, ethylene; MeJA, methyl jasmonate; 6-BA, 6-benzylaminopurine; MT, melatonin; NO, nitric oxide; nSe, nano-selenium.

Existing studies have preliminarily identified the transcription factors, phytohormones, and environmental stimuli that regulate *HCT/HQT* expression (Figure 5). However, the mechanistic links connecting these distinct regulatory layers remain poorly understood. The full signaling cascade—spanning initial environmental perception, phytohormone-mediated signal transduction, downstream transcription factor activation, and ultimately coordinated regulation of *HCT/HQT*—has yet to be systematically elucidated.

## 7. Conclusions and Prospects

### 7.1. Conclusions

HCT/HQT is a central enzyme in plant secondary metabolism, playing a pivotal role in the biosynthesis of lignin, CGA, and flavonoids through the phenylpropanoid pathway. These metabolites are indispensable for plant growth and development [29] and confer substantial resistance to biotic stresses—including pathogen infection—as well as enhanced resilience to abiotic challenges [64]. The three-dimensional structure of HCT/HQT has been determined, revealing a bilobal architecture composed of two similar-sized domains (Domain I and Domain II) connected by an extended cross-loop. Structural and biochemical studies have provided initial mechanistic insights, indicating that catalysis depends on dynamic enzyme–substrate interactions. Protein engineering strategies—including rational design and directed evolution—have successfully broadened substrate specificity and enhanced catalytic versatility, thereby expanding the structural diversity of downstream metabolites [110]. Functionally, HCT/HQT catalyzes the critical C3-hydroxylation step in the phenylpropanoid pathway and serves as a major flux-controlling node. Its activity directly regulates carbon partitioning among three competing metabolic branches: lignin monomer biosynthesis, CGA formation, and flavonoid production. Consequently, HCT/HQT modulates cell wall composition and properties—thereby enhancing mechanical strength, hydrophobicity, and resistance to oxidative stress and pathogen invasion—ultimately improving plant adaptability to complex environmental conditions. The expression of *HCT/HQT* is tightly coordinated by both endogenous regulators—including MYB, bHLH, and WRKY transcription factors—and exogenous cues such as light, temperature, and phytohormones, thereby forming an integrated regulatory network that fine-tunes phenylpropanoid metabolism in response to developmental and environmental signals.

The *HCT/HQT* gene family originated during land plant evolution, with orthologs first identified in mosses [111]. Its biochemical function has been highly conserved across both vascular and non-vascular plant lineages. As in higher plants, bryophytes utilize HCT/HQT enzymes in both direct and indirect capacities to support the biosynthesis of lignin-related polymers. Current research focuses predominantly on angiosperms, underscoring the multifaceted roles of these enzymes—not only in lignin, CGA, and flavonoids biosynthesis, but also in mediating abiotic and biotic stress tolerance. Deciphering the crosstalk and synergistic regulatory mechanisms among these interconnected metabolic branches is essential for understanding the systemic integrity and functional plasticity of plant secondary metabolism. These efforts will ultimately bridge fundamental insights into HCT/HQT biology with actionable targets for crop improvement, sustainable bioenergy production, and the development of next-generation stress-tolerant cultivars.

### 7.2. Prospects

Building upon the current understanding of HCT/HQT structure, function, and regulatory mechanisms, further investigations are needed to address key unresolved questions. In particular, comparative genomics, metabolic engineering, CRISPR-based functional characterization, and integrative multi-omics approaches represent promising avenues for future research.

Leveraging the rapidly expanding repository of plant genomic data, systematic phylogenomic analyses should be conducted to elucidate the evolutionary dynamics of the BAHD family across diverse plant lineages—specifically, to identify the phylogenetic nodes at which HQT enzymatic activity was lost in monocotyledonous plants. Integrating phylogenetic reconstruction with synteny analysis enables the identification of critical sites underlying functional divergence; such insights will support the development of predictive models that link sequence features to substrate selectivity, thereby facilitating the functional annotation of uncharacterized BAHD family members.

Metabolic engineering constitutes the core strategy for unlocking the application potential of HCT/HQT. In microbial systems, heterologous hosts must be systematically optimized via metabolic engineering to augment the intracellular availability of key precursors. Concurrently, directed evolution and rational design should be leveraged to enhance catalytic efficiency and substrate specificity, thereby facilitating the construction of high-performance microbial cell factories.

CRISPR-Cas9 technology should be harnessed to construct comprehensive mutant libraries encompassing *HCT/HQT* genes and their orthologs. This strategy enables systematic phenotypic characterization of both single- and multi-gene knockouts and facilitates the comparative analysis of functional divergence between monocots and dicots. Moreover, CRISPR activation (CRISPRa) and CRISPR interference (CRISPRi) systems should be developed to achieve reversible, tunable regulation of endogenous *HCT/HQT* expression—thereby permitting precise functional dissection in a tissue- or developmental stage-specific manner. When integrated with CRISPR-based screening approaches, these tools will support the identification of upstream transcription factors and signaling components that govern *HCT/HQT* expression, ultimately enabling the reconstruction of complete regulatory signaling cascades.

The complexity of the phenylpropanoid metabolic network indicates that single-level investigations are insufficient to elucidate its systemic regulatory mechanisms. Future research must therefore adopt integrative, multi-omics approaches. By integrating association analysis across genomic variation, transcriptomic profiles, and metabolite accumulation, predictive models that robustly link genotype to phenotype can be established. Machine learning algorithms provide a powerful computational framework for the integrative analysis of multi-omics data, enabling the identification of key regulatory nodes and feedback loops that govern HCT and HQT activity—thereby facilitating the precise prioritization of metabolic engineering targets. In conclusion, HCT/HQT functions as a central regulatory hub at the intersection of phenylpropanoid and flavonoid metabolism. Future research should employ rigorously integrated multi-omics approaches—encompassing genomics, transcriptomics, proteomics, and metabolomics—and complement them with systematic experimental validation to decipher the context-dependent regulatory logic governing HCT/HQT within plant metabolic networks. Such integrative efforts will not only advance our mechanistic understanding of its biological roles but also fully harness its potential for crop improvement, pharmaceutical discovery, and sustainable biomanufacturing.

## Data Availability

The original contributions presented in this study are included in the article. Further inquiries can be directed to the corresponding authors.

## Abbreviations

The following abbreviations are used in this manuscript:

HCT/HQT	Hydroxycinnamoyl-CoA shikimate/quinate hydroxycinnamoyl transferase
HCT/HST	Hydroxycinnamoyl-CoA shikimate hydroxycinnamoyl transferase
HQT	Hydroxycinnamoyl-CoA quinate hydroxycinnamoyl transferase
UV	Ultraviolet
BEAT	Benzyl alcohol O-acetyltransferase
AHCT	Anthocyanin O-hydroxycinnamoyl transferase
HCBT	Hydroxycinnamoyl/benzoyl transferase
DAT	Deacetyl vindoline 4-O-acetyltransferase
qRT-PCR	Quantitative reverse transcription PCR
CGA	Chlorogenic acid
MbA	Montbretin A
Asp	Aspartate
VS	Vinorine synthase
Thr	Threonine
Ser	Serine
Tyr	Tyrosine
His	Histidine
Arg	Arginine
Trp	Tryptophan
HS-CoA	Coenzyme A
Leu	Leucine
Phe	Phenylalanine
ABA	Abscisic acid
MeJA	Methyl jasmonate
GA_3_	Gibberellic acid 3
SA	Salicylic acid
6-BA	Cytokinin
MT	Melatonin
C3’H	P-coumaroyl ester 3’-hydroxylase
CHS	Chalcone synthase
CHI	Chalcone isomerase
PAL	Phenylalanine ammonia-lyase
C4H	Cinnamic acid-4-hydroxylase
HCAAs	Hydroxycinnamic acid amides
EC-CA	Epicatechin-3-O-caffeoate
WPE	Ethanol whole plant extract
PR-1	Pathogenesis-related protein-1
TMV	Tobacco mosaic virus
MOE	Modulus of elasticity
ALV	Application of aloe vera
4CL	4-coumarate-CoA ligase
TF	Transcription factor
VIGS	Virus-induced gene silencing
RNAi	RNA interference
GWAS	Genome-wide association study
eQTL	Expression quantitative trait loci
Eth	Ethylene
NO	Nitric oxide
nSe	Nano-selenium
